# Dr. Drake’s Introductory Lecture

**Published:** 1844-12

**Authors:** 


### DR. DRAKE'S INTRODUCTORY LECTURE.

Our Senior must pardon us for saying here a word about his Introductory Lecture. We did not have the pleasure of hearing it delivered, but we have derived great pleasure from its perusal. We are glad the class insisted on its publication—it is as creditable to them, as it is honorable to the professor. The subject of the lecture is, *The Means of Promoting the Intellectual Improvement of the Students and Physicians of the Valley of the Mississippi.*—It abounds in just views, expressed with the force and earnestness characteristic of the author, and ought to be read by every member of the profession in the South and West. We must quote the following passage, not as a specimen of the lecture, but for the sake of its *graphic truth.* The author is speaking of the principles which should guide the young student after having attained the doctorate:

"My firm opinion is, that one of the causes which retard us, in raising our profession in the West and South, to an excellence which at present seems almost hopeless, is the style in which our offices are fitted up and kept. Who can read and think, with method or sound logic, while everything around him is dirty and disordered? His little stock of furniture displaced, as if a riot had just passed away; his books scattered on chairs, tables, and the greasy medicine shelves; in his book-cases, volumes of different sets mixed together, some lying flat, and some, like the ideas of their reader, up side down; his skeleton exposed, and joint after joint torn off; his few injected preparations, unvarnished as my narrative, and worm-eaten as the books of an old doctor; his medicines unlabelled, and thrown into a chaos, as great as a treatise on the Materia Medica in the 14th century; bundles untied, and bottles left uncorked, or stopped with plugs of paper; dead flies in the ointment within his jars, while others are wading through that which has lain so long spread over his counter, that their feet are blistered by its rancidity; his spatulas foul and rusty; his scales tied with strings and balanced with pieces of paper; his mortar about as clean as the ancient Kentucky hommony block, which, in the same day, contained the food of the family, and of the family cow and horse, as it stood convenient to all the parties, on neutral ground, near the door of the cabin; his surgical instruments oxidated and rusting away, like his mind; his study table, covered with loose papers and medical journals (even the Western) with their covers torn off; his walls overspread with a tapestry of cobwebs; his windows as opake from the dust as the painted glass of an ancient cathedral; his foul candlestick standing all day on his lexicon, and his floor spotted over with the blood of his surgical patients, and his own tobacco juice!

The human intellect cannot act when thus encompassed. Ideas will not arrange themselves; nor will their foul surfaces cohere. The scene re-acts upon his mind, and a chaos within rivals that without." C.

